# A Ferrofluid with High Specific Absorption Rate Prepared in a Single Step Using a Biopolymer

**DOI:** 10.3390/ma15030788

**Published:** 2022-01-20

**Authors:** Dulce A. Guzmán-Rocha, Teodoro Córdova-Fraga, José J. Bernal-Alvarado, Zaira López, Francisco A. Cholico, Luis H. Quintero, José A. Paz, Mario E. Cano

**Affiliations:** 1 Departamento de Ingeniería Física, División de Ciencias e Ingenierías, Universidad de Guanajuato, Campus León, Loma del Bosque 103, Lomas del Campestre, León 37150, Mexico; dul_ara@yahoo.com.mx (D.A.G.-R.); theo@fisica.ugto.mx (T.C.-F.); bernal@ugto.mx (J.J.B.-A.); 2 División de Desarrollo Biotecnológico, Centro Universitario de la Ciénega, Universidad de Guadalajara, Av Universidad 1115, Col. Linda Vista, Ocotlan 47820, Mexico; zlopez@gmx.net (Z.L.); pako.cholyko@gmail.com (F.A.C.); jocmos@hotmail.com (J.A.P.); 3 Departamento de Economía, Centro Universitario de Ciencias Económico-Administrativas, Universidad de Guadalajara, Periférico Norte N° 799 Núcleo Universitario, Los Belenes, Zapopan 45100, Mexico; hectorquintero@yahoo.com

**Keywords:** arabic gum, magnetic saturation, iron oxide, hyperthermia

## Abstract

An exhaustive characterization of the physicochemical properties of gum arabic (GA)-coated Fe_3_O_4_ magnetic nanoparticles was conducted in this work. These nanoparticles were obtained via the in-situ coprecipitation method (a fast single-step method) in two GA:Fe ratios, 10:1 and 20:1, respectively. Several experimental techniques were applied in the characterization process, all of them described below. Using Transmission Electron Microcopy images, they were shown to have spherical-like morphology with 11 nm diameter. The Fourier Transform Infrared spectra confirmed the attachment of the GA on the surface of the magnetic nanoparticles (MNPs), providing good colloidal stability from pH 7 to 8. The thickness of the coatings (1.7 nm and 1.1 nm) was determined using thermogravimetric measurements. A high specific absorption rate and superparamagnetic properties were determined using alternant and static magnetic fields, respectively. The GA-coated MNPs were non-cytotoxic, according to tests on HT-29 human intestine cells. Additionally, HT-29 cells were exposed to magnetic fluid hyperthermia at 530 kHz, and the induction of cell death by the magnetic field, due to the heating of GA-coated MNP, was observed.

## 1. Introduction

The use of nanostructured materials, such as nanoparticles (NPs), offers many interesting physical properties, which contrast with the observations of bulky materials [1]. For example, NPs have a high surface-to-volume ratio that provides extremely reactive and versatile properties. Magnetic nanoparticles (MNPs) have found numerous applications in biomedicine fields, such as magnetophoresis, drug delivery, nuclear magnetic resonance imaging (NMRI), and hyperthermia [2,3]. Additionally, MNPs can be manipulated by high gradients of magnetic fields [1].

In the scope of the physicochemical area, the biocompatibility of MNPs is directly related to particle size, shape, hydrophilic nature, coating, and surface charge [4,5]. All these properties are fundamental to carrying out trials of magnetic fluid hyperthermia (MFH), where the implantation of localized iron oxide MNPs helps to concentrate the energy in a chosen biologic tissue [6], where the temperature can be increased above 42–43 °C using an AC magnetic field [7].

On the other hand, gum arabic (GA) is a biopolymer structurally composed of three molecular fractions, the arabinogalactan-peptide (AGp), arabinogalactan-protein (AGP), and glycoprotein (GP) complexes [8], and has been used in nanotechnology applications in both in vitro and in vivo assays. GA helps to stabilize MNP clusters while maintaining their physicochemical properties and increasing their biological compatibility. These characteristics have shed light on its potential usage for molecular drug delivery, being capable of improving the aqueous stability of NPs [9]. Additionally, GA has been studied as a possible coating for MNPs, providing reactive functional groups suitable for the coupling of bioactive compounds [10]. It is known [11] that a coating of polyarabic acid on MNPs promotes their internalization in breast cancer cells. These core-shell structures are also appropriate to be used as a contrast medium in NMRI. As far as we know, few works have been published on the suitability of using GA-coated MNPs for MFH using cell lines and electromagnetic induction heaters. A new work [12] reported the effect of GA on the increase in the specific absorption rate SAR of Fe_3_O_4_ MNPs, which is one of the most important parameters in MFH. This effect is due to the increment of their heating rate and the diminution of the specific heat provided by GA.

Typically, the synthesis of MNPs is based on the coprecipitation method. MNPs firstly suspended in water are subsequently coated by ultrasonication for a predefined time. In this research, the synthesis of MNPs and their coating with GA is performed in a single step (in situ GA coating), making the preparation procedure more efficient. This is in agreement with the report [13] where the following effect was observed: adsorption on the nuclei and the growing crystals can inhibit the growth of the particles, promoting the formation of small units. This work studies the effect of the attachment of a GA shell on the surface of the iron oxide MNP; to this end, two different concentrations of GA were added to modify the thickness of the coating, performing a single-step synthesis. Additionally, the physicochemical properties of the coated MNP and its suitability to be used in MFH in vitro experiments were analyzed, particularly using the human colorectal cell-line HT-29. As will be shown, ferrofluids meet the majority of the ideal requirements for biological applications summarized in [14]. In several scientific reports, the physicochemical and cytotoxic properties of very similar ferrofluids have been analyzed to determine the effects of GA coating, reporting its suitability for use in a number of biomedical applications. Nevertheless, these works are lacking in vitro experiments on MHT with mammalian cancer cells.

## 2. Materials and Methods

### 2.1. Materials

The basic materials to perform the experiments were purchased from Sigma-Aldrich (Madrid, Spain): iron(II) chloride tetrahydrate reagent grade 98%, iron(III) chloride hexahydrated with a purity of 97%, gum arabic from acacia tree branched polysaccharide, and ammonium hydroxide ACS reagent 28–30%, analytical grade.

### 2.2. In Situ Synthesis of GA-Coated MNPs

Two samples named MNP-GA1 and MNP-GA2 were prepared, using the coprecipitation method of MNPs and in situ coating of MNPs with GA. The same methodology was followed for the two kinds of samples, changing only the amount of GA, as is explained in the next separated stages.

- Initially, in two separated flasks, 11 mL of FeCl_3_·6H_2_O 0.4 M and 11 mL of FeCl_2_·4H_2_O 0.2 M were dissolved in 100 mL of distilled water.
- In the first flask, 10 mL of 10% *w*/*w* GA (for MNP-GA1) was added, and in the second, 10 mL of 5% *w*/*w* GA (for MNP-GA2).
- To each solution obtained in each flask, 20 mL 0.3 M solution of (25%) NH_4_OH was slowly added; then, these mixtures were stirred at 380 rpm and heated for 90 min at 90 °C.
- The coated MNPs of the obtained solutions were separated by magnetic decantation using a large permanent magnet (1.2 T in the surface) and adding 80 mL of distilled water. This washing procedure was repeated three times.
- Finally, the MNPs were suspended in water obtaining two ferrofluids with GA-coated MNPs.

During the synthesis, N_2_ inert atmosphere was used to avoid the oxidation of the MNPs. It is important to highlight the rapid procedure followed, where several steps of sonication and washing were omitted, contrasting with the typical synthesis method used to produce core-shell structures. To obtain uncoated MNPs, a second preparation was carried out following the same steps above, omitting the solution of GA; in this case, it obtained a ferrofluid of Fe_3_O_4_ suspended in water.

### 2.3. Characterization Techniques

The crystalline structures of MNP-GA1 and MNP-GA2 were studied by X-ray diffraction (XRD), performed in a Bruker D8 Advance Powder Diffractometer (Karlsruhe, Germany) using Cu Kα radiation. Previously, aliquots of all the ferrofluids were dried on a stove at 60 °C, and samples with 10 mg of powder were placed in silicon plates, then the patterns were collected between 10° and 90°. Particle sizes and shapes were determined by Transmission Electron Microscopy (TEM), using a Microscope JEOL model JEM 1010 (Tokyo, Japan) complemented with a digital camera ORIUS (Gatan^®^, Berwyn, PA, USA). Thus, aliquots of each ferrofluid were diluted at 1:100 and deposited onto FCF-200-Cu grids for drying at room temperature within an inert atmosphere, and TEM micrographs (at 80 kV, 250–300 kx of magnification) were registered and stored. The distribution and the mean particle size were evaluated by measuring the largest internal dimension of at least 200 particles, and Origin-MicroCal (Version 6.0, Northampton, MA, USA) was used for data processing. To verify the attaching of GA on the surface of magnetite, Fourier Transform Infrared (FTIR) spectroscopy was used, and the spectra were recorded between 4000 and 400 cm^−1^ in a Bruker Vertex 70V spectrometer (Ettlingen, Germany). For this purpose, powdered samples were prepared by dilution of the dried uncoated and coated MNPs (2 wt.%) in KBr, and compression of the mixture into special pellets. The thickness of the coating of GA was determined by Thermogravimetric (TGA) analysis using the SDT Q600 instrument (TA instruments, Leatherhead, Surrey, UK). Then an initial mass of each dried sample was heated from room temperature up to 900 °C while its weight was registered, following a ramp of 10 °C/min under an airflow rate of 100 mL/min. Colloidal properties of the samples were studied in a Zeta-sizer Nano S (Malvern Instruments, Malvern, UK). The colloidal stability of the ferrofluids with MNP-GA1 and MNP-GA2 was measured for several pH by determining their Z-potential. Subsequently, the hydrodynamic size of the particles was determined (pH ≈ 7.4) by Dynamic Light Scattering (DLS) (Malvern Instruments, Malvern, UK). Then, the special capillary cells were filled with aliquots of each ferrofluid, which were mixed with drops of 0.1M NaOH to modify the pH over the interval from pH = 2 to 10.

A vibrating sample magnetometer (VSM) of Quantum Design (San Diego, CA, USA) was used to measure the magnetic properties of the MNP-GA1 and MNP-GA2 at room temperature. Then, dried powdered samples were accurately weighed and pressed into a sample holder. Hysteresis loops were measured at room temperature over the interval −2387 kAm^−1^ < H < 2387 kAm^−1^. Furthermore, the zero-field-cooled/field-cooled (ZFC-FC) traces were registered by fixing H = 7.96 kAm^−1^ and covering the temperature interval from 50 K to 400 K. Additionally, to determine the response of the ferrofluid with MNP-GA2 under AC magnetic fields, a frequency variable induction heating system [15,16,17] was employed. For these estimations, three samples of 1 mL were deposited into 2 mL Eppendorf tubes, which were placed within the cavity of the magnetic field generator. The mass fraction of suspended MNPs in the samples was η = 0.1%, and f = 530 kHz was the frequency of the magnetic field irradiation, with intensities covering the interval 12.5 kAm^−1^ < H < 25 kAm^−1^ (using steps of 4.17 kAm^−1^). Additionally, a sample with 1 mL of water (without nanoparticles) was used to determine the background of the heating not related to the magnetic field. In all the measurements, a fluoroptic sensor Luxtron-One (Santa Clara, CA, USA) was used to obtain the temperature of the samples during the magnetic field irradiation.

### 2.4. Cell Line and Culturing Conditions

The cell line HT-29 was purchased from ATCC (Manassas, VA, USA). The HT-29 cell line was cultivated in DMEM (ATCC) supplemented with 10% FBS (Biowest, Kansas City, MO, USA) and 10 mM HEPES (pH = 7.3, Promega Madison, WI, USA). The conditions of incubation were 37 °C, 4% CO_2_, and 95% RH. Assays were carried out in 12-well microtiter plates (MTP) or single vials. Cover slides were placed on them when necessary, and 1 mL of DMEM with 50 µg/mL of gentamicin was then added. A total of 4 × 10^4^ cells were inoculated for 24 h to let them attach.

### 2.5. Cytotoxicity and Magnetic Fluid Hyperthermia (MFH) Assays

After 24 h of incubation, the cells had adhered and the old medium was removed, and the new medium with MNP-GA2 ferrofluid was added. For the cytotoxicity assay, the concentrations were 0.5, 1.0, 2.0, and 3 mg/mL, and the cells were again incubated for 24 h. For the MFH assay, the concentration of MNP-GA2 was 2.0 mg/mL and the cells were incubated for only 2 h, which allowed the internalization of nanoparticles into the cells. Subsequently, the cells were exposed to MFH for 20 min at temperatures of 37, 39, 42, 45, and 47 °C (± 0.5 °C); after the heat treatment, the cells were incubated 24 h. After incubation, the old medium was removed and the cells were washed twice with phosphate-buffered saline (PBS).

In order to determine the metabolic activity, quantitative (WST-1) and qualitative (neutral red uptake and trypan blue) analyses were performed. Thus, 20 µL of WST-1 (Clontech, Mountain View, CA, USA) and 1 mL of new medium were added to the cells, and were incubated for a further 4 h. After that time, the mixture was centrifuged (1 min, 10,000 rpm; Eppendorf 5415 D, Hamburg, Germany) before measurement of the absorbance (Biopop, Mecasys, Korea) at λ 440 nm and λ 690 nm. The remaining cells were incubated for 4 h further, with 1 mL DMEM. For red staining, 30 µL at 0.33% of Neutral Red solution (Santa Cruz, Santa Cruz, CA, USA) was added. For blue staining, 10 µL at 0.4% of trypan blue (Biowest, River Side, MO, USA) was added. Subsequently, the red-stained samples were incubated for 2 h and then blue-stained for 10 min. Finally, the cover slides were observed under the microscope (Axioskop 40 FL, Zeiss, Oberkochen, Germany). All tests were done in triplicate.

## 3. Results and Discussion

### 3.1. PhysicoChemical Characterization of the GA-Coated MNPs

**Figure 1:**
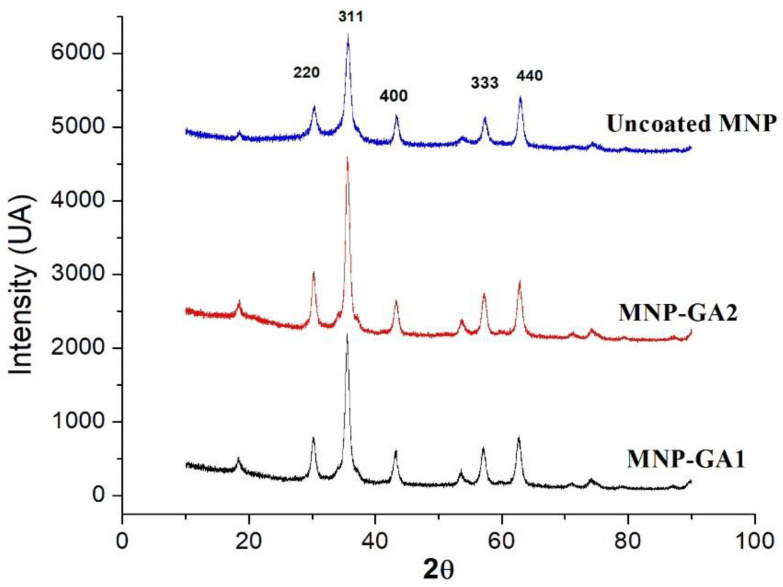
XRD patterns of the hybrid magnetic nanoparticles: MNP-GA1 (dark line), MNP-GA2 (red line) and uncoated MNPs (blue line).

The influence of the shell of GA on the crystalline structure of an MNP was studied using XRD (see Figure 1). The uncoated MNPs show (top spectrum) diffraction peaks at 30.2°, 35.7°, 43.4°, 57.4°, and 62.9° associated with the respective diffraction planes (220), (311), (400), (333), and (440), which correspond to the face-centered cubic structure of Fe_3_O_4_ [18]. As is explained in [19], the XRD technique must be complemented (in further experiments) with Mössbauer spectroscopy to determine the presence of maghemite in the samples. In the same figure, the diffractogram of MNP-GA1 and MNP-GA2 are plotted with the dark and red lines, respectively, and both spectra exhibit the same peaks as the uncoated MNPs. In each spectrum, the crystal size D is computed using the most intense peak corresponding to the plane (311), through the well-known Scherrer equation:

D = 0.89λ/βCosθ(1)

where β is the line broadening at half the maximum intensity and λ = 0.154 nm of wavelength is used. The values estimated for D are 10.2 nm, 10.8 nm, and 10.6 nm for uncoated MNPs, MNP-GA1, and MNPGA2, respectively. Thus, the peaks and sizes of D show the non-modification of the Fe_3_O_4_ structure when incorporated into a layer of GA.

**Figure 2:**
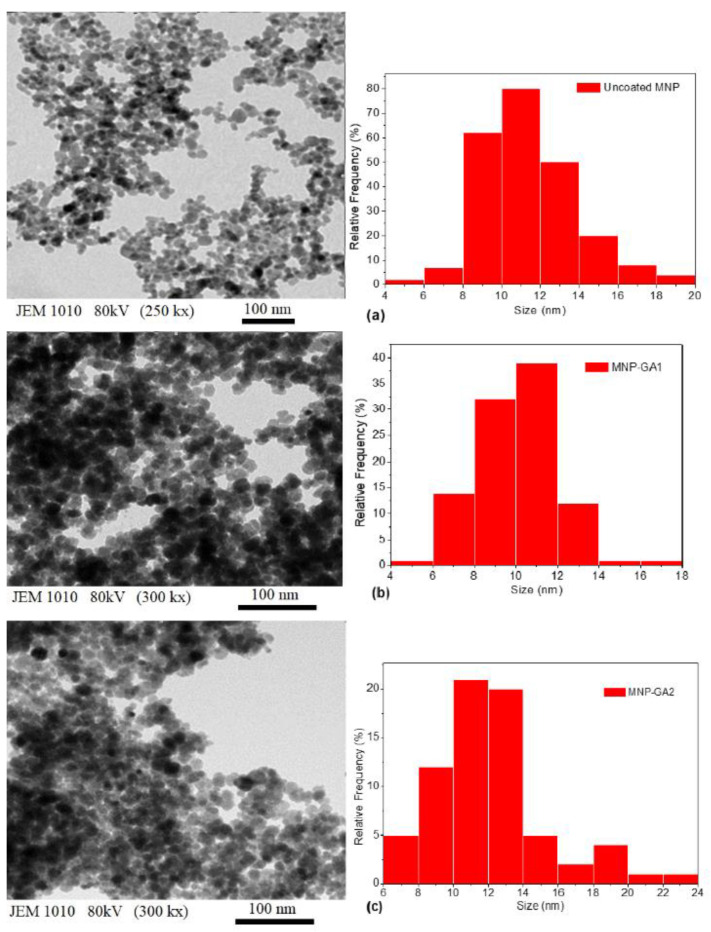
Typical TEM-images (acceleration voltage 80 kV with 250 and 300 kx of magnification) of the magnetic nanoparticles and their size distribution of (**a**) Uncoated MNPs, (**b**) MNP-GA1, and (**c**) MNP-GA2.

The size distribution and morphology of the uncoated MNPs, MNP-GA1, and MNP-GA2 were determined by TEM. Figure 2a–c shows TEM-micrographs, mainly showing a spherical morphology and almost homogeneous size distribution. A careful analysis of 200 nanoparticles allows a graphical description (bar plots) that shows a distribution centered very close to σ_C_ = 11 nm, with up to 1 nm of standard deviation (in round numbers). The width of the GA shell covering the uncoated MNP was not observed through TEM, probably because the resolution of TEM is not sufficient, and also because GA could be decomposed by the electron beam.

**Figure 3:**
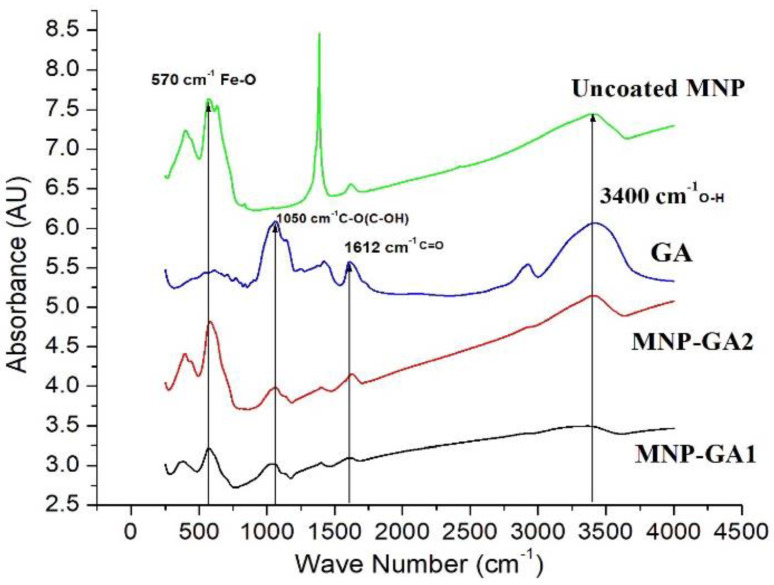
FT-IR spectra of the Fe_3_O_4_ MNPs (green line), GA (blue line), MNP-GA2 (red line), MNP-GA1 (dark line).

The FTIR technique was used to investigate the GA-MNP binding and to verify the presence of the main functional groups of the GA coating. Figure 3 shows the spectra of GA (blue line), MNP-GA1 (dark line), MNP-GA2 (red line), and uncoated MNPs (green line). In the GA spectrum, the following vibrations of its functional groups can be detected: at 1050 cm^−1^ corresponding to the torsional vibration of the CO(OH) bond; at 1612 cm^−1^ is associated with the C=O bond; around 3400 cm^−1^ the band is associated with the OH bond. In MNP-GA1(dark line) and MNP-GA2 (red line), the peaks corresponding to the functional groups of GA can be observed, as well as at 570 cm^−1^ corresponding to the Fe-O bond [20], which is characteristic of iron oxides. The bands, corresponding to magnetite, are in the region of low frequencies in the range of 400–1000 cm^−1^ [21]. Analyzing the absorbance of uncoated MNPs, the Fe-O stretching mode of the tetrahedral site in Fe_3_O_4_ is observed at 570 cm^−1^, and at around 1400 cm^−1^ a peak is registered related to the C-O stretching [22].

**Figure 4:**
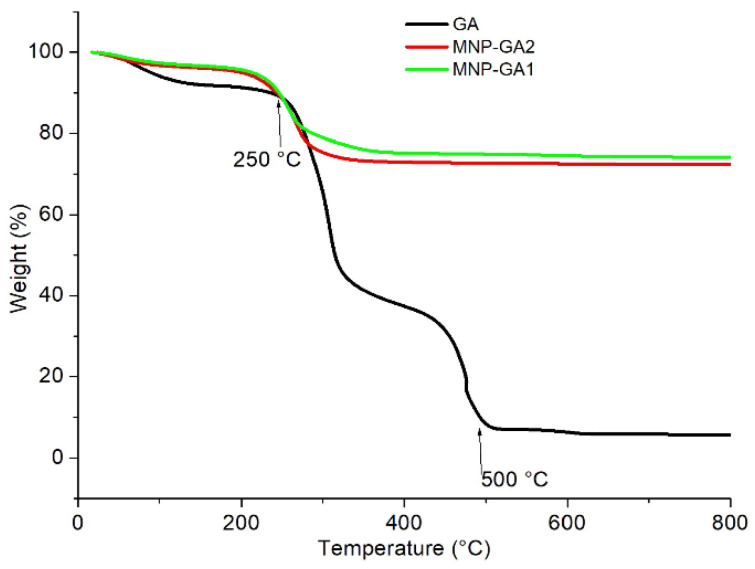
The relative dependence of the temperature on the mass loss of the GA (dark line), MNP-GA1 (green line), and MNP-GA2 (red line).

The results of the TGA analysis are shown in Figure 4. The relative mass of GA (dark line) shows a change in the slope close to its decomposition point of 250 °C, and it is almost completely decomposed above 500 °C. The same behavior is observed in the GA attached to magnetite. In MNP-GA1 (green line) the weight of GA decomposed by 25%, and in MNP-GA2 (red line) only 16% was decomposed. As both samples were washed several times, it is reasonable to assume that there was no GA dissolved in water and all the mass loss corresponded to decomposed GA, which was forming a shell on the core of magnetite. Thus, the thickness Δ of GA can be computed by applying the next deduced equation:

(2) $$\Delta =\frac{\sigma_{C}}{2}\left[{\left(\frac{\eta_{C}\rho_{S}}{\eta_{C}\rho_{S}+\eta_{S}\rho_{C}}\right)}^{-1/3}-1\right]$$

where (*η_C_*, *η_S_*) and (*ρ_C_*, *ρ_S_*) are the corresponding fractions of diminished mass and densities of the core Fe_3_O_4_ and shell of GA, respectively. Using the parameters *η_C_* = 0.75 and *η_S_* = 0.25 obtained from Figure 4) for MNP-GA1, as well as *ρ_C_* = 5.18 g/cm^3^ and *ρ_S_* = 1.4 g/cm^3^ as the densities of Fe_3_O_4_ and GA, respectively, it is estimated that ΔMNP-GA1 = 1.7 nm. In the same way, using *η_C_* = 0.84 and *η_S_* = 0.16, the thickness for MNP-GA2 is ΔMNP-GA2 = 1.1 nm.

**Figure 5:**
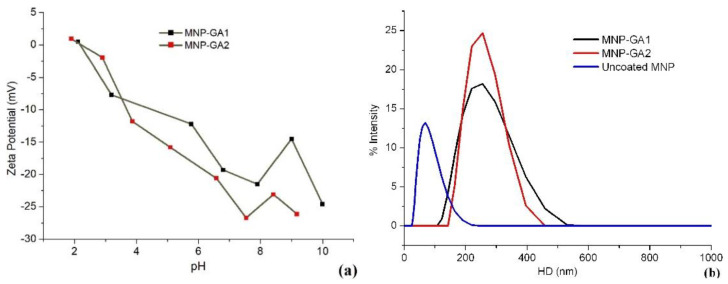
(**a**) pH-dependence of the zeta potential of MNP-GA1 and MNP-GA2 and (**b**) the corresponding hydrodynamic diameters of MNP-GA1 (at pH = 7.7) and MNP-GA2 (at pH = 7.5).

Zeta potential measurements were made in the pH range of 2 to 10, and the experimental results are displayed in Figure 5a. As can be observed, for MNP-GA1 the isoelectric point is reached at pH = 2.1, while for MNP-GA2 it is close to pH 2.6. Additionally, at pH = 7.7 and pH = 7.5, the respective zeta potential values are −21.5 mV for MNP-GA1 and −26.8 mV for MNP-GA2; these two intensities are evidence of the good colloidal stability of both ferrofluids in a physiological pH range. In the same sense, Figure 5b shows the hydrodynamic diameter HD of the uncoated and coated samples with pH ≈ 7.4. For the uncoated MNPs, it is observed that HD = 68 nm, while for MNP-GA1, HD = 245 nm with 0.17 of polydispersity index PDI, and for MNP-GA2, HD = 249 nm was reached, with 0.16 of PDI. Thus, the HD of the nanoparticles increased due to the GA coupling or thickness. This behavior of the HD indicates the presence of soft agglomerates, similar to islands of MNPs. This difference between the HD of coated and uncoated MNPs has been explained in [13,23], being associated with the interaction of GA through adsorption of carboxyl and hydroxyl groups on the surface of the magnetic nanoparticles. In another work [24], this increment of the HD was related to the formation of bridges between one GA molecule and more than one particle, especially when the coating was carried out in situ. Additionally, the lower magnetic remanence observed by VSM, and the relatively high thickness (about 22–28% of the average radius of coated MNPs) observed by TGA, led us to discard agglomerations by possible magnetic dipolar interparticle interactions.

**Figure 6:**
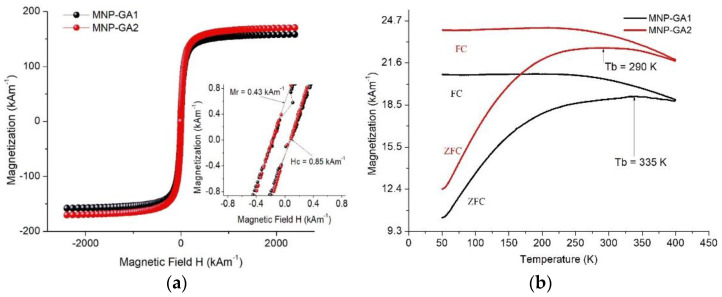
(**a**). The magnetization loop of the coated samples at room temperature, over the interval −2387 kAm^−1^ < H < 2387 kAm^−1^ including an inset plot. (**b**) Magnetization following the ZFC-FC trace with H = 7.96 kAm^−1^ over the interval from 50 K to 400 K.

The magnetization of the MNPs is shown in Figure 6a, where the magnetic parameters of magnetic saturation (Ms), coercivity (Hc), and magnetic remanence (Mr) can be observed (see the plot inset). The respective parameters reached are: for MNP-GA1 Mr_1_ = 158, Hc_1_ = 0.85 and Mr_1_ = 0.43 kAm^−1^, and Mr_2_ = 170, Hc_2_ = 0.85 and Mr_3_ = 0.43 kAm^−1^ for MNP-GA2. The small area within the hysteresis loop suggests principally the possible superparamagnetic behavior of the MNPs, but the small Hc exhibits the typical ferrimagnetic ordering. Additionally, the traces ZFC-FC (at H = 7.96 kAm^−1^) represented in Figure 6b show the blocking temperature for MNP-GA1 and MNP-GA2, reaching Tb = 335 K and 290 K, respectively, which are obtained by determining the maximum magnetization values of the ZFC traces. The existence of Tb is evidence of superparamagnetic behavior in both samples, which is expected in iron oxide MNPs with diameters less than 25 nm [25,26,27]. In particular, the MNP-GA2 sample is superparamagnetic even at room temperature, which is a property desirable for MFH applications because it diminishes the risk of vessel embolization (due to the sizes of the MNPs), and the particles maintain null dipolar interactions with a high SAR [26]. A careful observation of the FC traces shows a diminution of the magnetization (below 185 K) only in the MNP-GA2 sample; this evidence of the weak dipolar interactions of the nanoparticles, which is associated with the smaller thickness reached of GA ΔMNP-GA2. Thus, the mixed superparamagnetic and weak ferromagnetic behaviors observed are due to the small polydispersity of the MNP diameters, and this phenomenon is consistent with the slow increase in the wide ZFC trace.

**Figure 7:**
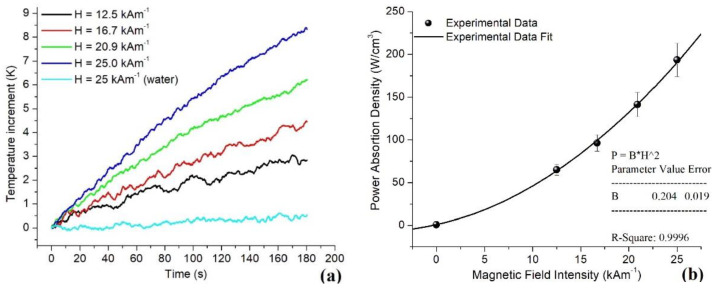
(**a**). Time dependence of the temperature rise of MNP-GA2, using several intensities of H over the interval 12.5 kAm^−1^ < H < 25 kAm^−1^, with 4.17 kAm^−1^ increments. One measurement at H = 25 kAm^−1^ corresponds to a reference sample with 1 mL of pure water. (**b**) Estimation of P in W/cm^3^ of the ferrofluid with MNP-GA2.

The calorimetric measurements were performed on MNP-GA2 because it exhibited the highest colloidal stability and lower Tb. Thus, this sample was superparamagnetic in a typical temperature interval needed for MFH experiments, 37 °C < T < 46 °C. The rise of the ferrofluid temperature over time is displayed in Figure 7a. As can be observed, the maximum temperatures reached increased with the increment of H, while the heating of the water sample was negligible (approximately 1.7 mK/s). Subsequently, the slope dT/dt of all the curves was computed over the lapse 50 s < t < 100 s, and the power absorption density P was estimated using the equation:

P = *ρ*_FF_(η^−1^)(c_v_)(dT/dt) (3)

The corresponding values are depicted in Figure 7b. Due to the small value of η, the parameter P involves *ρ*_FF_ = 1.0 g/cm^3^ and c_v_ = 4.18 J/(kg × K) which are the same as water. Additionally, Figure 7b shows the regression of the experimental measurements, fitting the mathematical expression P = B × H^2^ and getting B = 0.2 W/(cm^3^Am^−1^)^2^ as the proportionality constant (with R^2^ = 0.999 of correlation). These results led to the following prediction: 1 mg of the MNP-GA2 is capable of dissipating up to 200 W per cm^3^ in cancerous tissue. The SAR of the sample can be estimated by dividing P/*ρ*_FF_ ≈ 200 W/g, which is a larger capacity than that observed in other ferrofluids with high SAR used in vitro and in vivo experiments [28,29].

### 3.2. Cell Biological Experiments

**Figure 8:**
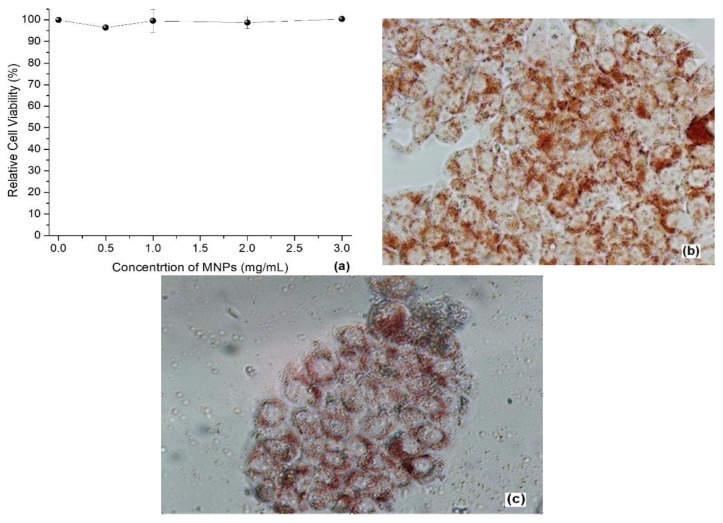
(**a**) Metabolic activity of HT-29 cells quantified as the relative WST activity when they were exposed to MNP-GA2 ferrofluid. Growth control activity was set to 100% for each experiment. Neutral red uptake of HT-29 after 24 h exposure to MPN-GA2: (**b**) Growth control (10× magnification); (**c**) cells exposed to γ = 3.0 mg/mL of MPN-GA2 (40× magnification).

The relative viability of HT-29 cells exposed to different concentrations γ of MNP-GA2 is shown in Figure 8a. This quantitative experiment exhibits the absence of cytotoxicity even with γ = 3.0 mg/mL of MNP-GA2. These results were confirmed qualitatively by neutral red uptake (NRU), where functional lysosomes were red-stained. In all concentrations, HT-29 cells were red-stained (see for example the control cells in Figure 8b and the cells exposed at γ = 3 mg/mL in Figure 8c), and furthermore, they had regular and homogenous shape, functional lysosomes, membranes, and confluence between 80%–90%. As can be expected, MNP-GA2 was nontoxic, because GA is an edible polysaccharide with vigorous water solubility, which is recognized as safe (GRAS) to be used as emulsifying and thickening in the food industry [30].

**Figure 9:**
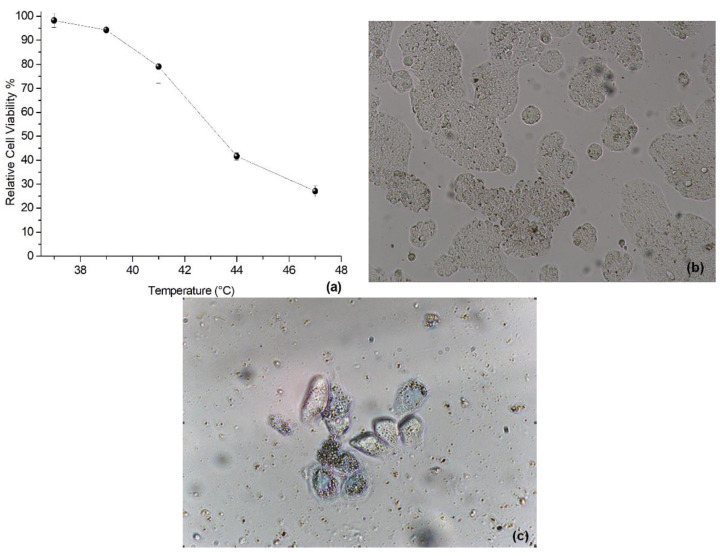
(**a**) Cytotoxic effect on HT-29 cells when they were exposed to 2.0 mg/mL NMP-GA and MFH at different temperatures. Cells stained with trypan blue after the exposition to MNP-GA2, 2.0 mg/mL, and MFH at different temperatures: (**b**) Growth control at 37 °C (10× magnification) and (**c**) Cells heated at 47 °C (40× magnification).

HT-29 cells were exposed to γ = 2.0 mg/mL of MNP-GA2 and summited to MFH treatment at 37, 39, 41, 43, and 47 °C. Metabolic activity of the cells slightly decreased from 37 to 41 °C, nevertheless, it was dramatically diminished (Figure 9a) at 44 and 47 °C, reaching up to 41.7% and 27.13% of confluence, respectively. Additionally, a few cells were blue-stained and also had rounded shapes, the confluence remaining around 60–70% at temperatures of 39–41 °C (Figure 9b). Meanwhile, some necrotic cells with disrupted membranes and irregular shapes were observed (most of them were blue-stained) at 44 and 47 °C (Figure 9c), reaching confluence values between 5–10%. These findings indicate that MFH treatment promotes the death of cells due to the heating of the MNP-GA2.

The surface charge of nanoparticles influences the cellular internalization and trafficking pathways, which can be clathrin-mediated endocytosis (CME) and caveloin-mediated endocytosis (CVME). The paramagnetic properties of iron oxide nanoparticles allow them to be directed to specific areas using external magnetic fields; thus, they can enter the cells by CVME. It is known that nanoparticles decorated with biomaterials, such as PEGylated substances, use this same pathway [31]. The internalization of nanoparticles into cells promotes several physiological effects; nevertheless, the effects of MNP-GA2 combined with MFH are not broadly known. It has been shown that MNP-GA in 9L glioma cells had significant cellular uptake. These findings were confirmed by magnetic resonance imaging where the accumulation of MNP-GA was observed at a tumor site in rats harboring 9L glioma tumors [30]. MFH treatment combined with MNPs causes a little-known effect: the release of ROS, which can sensitize cancer cells to make them vulnerable to subsequent apoptotic magnetic hyperthermia at 43 °C [32].

## 4. Conclusions

Two ferrofluids of iron oxide MNPs covered with a biopolymeric coating of GA were synthesized in a single step. The thickness Δ of GA depends on the Fe:GA ratio, particularly. When the ratios were 10:1 (MNP-GA1) and 20:1 (MNP-GA2), Δ was reached at approximately 1.1 nm and 1.7 nm, respectively. Good colloidal stability was observed in aqueous suspension for MNP-GA1 and MNP-GA2, and this last exhibits a slightly higher hydrodynamic diameter. The magnetic characterization indicated that both core-shell structures present superparamagnetic properties, with blocking temperatures Tb of 335 K and 290 K, respectively. Because MNP-GA2 exhibits lower Tb and higher zeta potential, it was selected to carry out magnetocalorimetric experiments and magnetic hyperthermia assays. This sample was capable of producing a power loss of up to 200 W/cm^3^, or a high SAR compared with other ferrofluids used in vitro or in vivo experiments. Cytotoxicity assays on HT-29 cell line showed that MNP-GA2 was not toxic, even when the cells were exposed to up to 3 mg/mL of nanoparticles. Moreover, the MFH assays showed a good effectivity for destroying mammalian cancer cells using AC magnetic fields of 530 kHz, because of the high-power absorption density of the coated nanoparticles provided by the GA.

The great advantages observed using GA as a coating for MNPs are biocompatibility and economy in the synthesis process, in addition to the fact that the entire process is environmentally friendly. Concerning the incorporation of magnetite into GA, which was carried out in situ, it was confirmed that GA does not interfere with the size of the crystal when it is directly added to the components of the reaction. In summary, MNP-GA2 meets the requirements for biological applications, with the qualities of non-aggregation, water compatibility, protection and stability of the MNP, as well as biocompatibility and no toxicity.

## Data Availability

The data presented in this study are available on request from the corresponding author.
